# The Feasibility and Acceptability of a Web-Based Alcohol Management Intervention in Community Sports Clubs: A Cross-Sectional Study

**DOI:** 10.2196/resprot.6859

**Published:** 2017-06-30

**Authors:** Tameka McFadyen, Luke Wolfenden, John Wiggers, Jenny Tindall, Sze Lin Yoong, Christophe Lecathelinais, Karen Gillham, Shauna Sherker, Bosco Rowland, Nicola McLaren, Melanie Kingsland

**Affiliations:** ^1^ School of Medicine and Public Health Faculty of Health The University of Newcastle Wallsend Australia; ^2^ Hunter New England Population Health Hunter New England Local Health District NSW Health Wallsend Australia; ^3^ Australian Drug Foundation Melbourne Australia; ^4^ Deakin University Melbourne Australia

**Keywords:** alcohol, sports, implementation, technology, TAM, Web, Internet, eHealth

## Abstract

**Background:**

The implementation of comprehensive alcohol management strategies can reduce excessive alcohol use and reduce the risk of alcohol-related harm at sporting venues. Supporting sports venues to implement alcohol management strategies via the Web may represent an effective and efficient means of reducing harm caused by alcohol in this setting. However, the feasibility and acceptability of such an approach is unknown.

**Objective:**

This study aimed to identify (1) the current access to and use of the Web and electronic devices by sports clubs; (2) the perceived usefulness, ease of use, and intention to use a Web-based program to support implementation of alcohol management policies in sports clubs; (3) the factors associated with intention to use such a Web-based support program; and (4) the specific features of such a program that sports clubs would find useful.

**Methods:**

A cross-sectional survey was conducted with club administrators of community football clubs in the state of New South Wales, Australia. Perceived usefulness, ease of use and intention to use a hypothetical Web-based alcohol management support program was assessed using the validated Technology Acceptance Model (TAM) instrument. Associations between intention to use a Web-based program and club characteristics as well as perceived ease of use and usefulness was tested using Fisher’s exact test and represented using relative risk (RR) for high intention to use the program.

**Results:**

Of the 73 football clubs that were approached to participate in the study, 63 consented to participate and 46 were eligible and completed the survey. All participants reported having access to the Web and 98% reported current use of electronic devices (eg, computers, iPads/tablets, smartphones, laptops, televisions, and smartboards). Mean scores (out of a possible 7) for the TAM constructs were high for intention to use (mean 6.25, SD 0.87), perceived ease of use (mean 6.00, SD 0.99), and perceived usefulness (mean 6.17, SD 0.85). Intention to use the Web-based alcohol management program was significantly associated with perceived ease of use (*P*=.02, RR 1.4, CI 1.0-2.9), perceived usefulness (*P*=.03, RR 1.5, CI 1.0-6.8) and club size (*P*=.02, RR 0.8, CI 0.5-0.9). The most useful features of such a program included the perceived ability to complete program requirements within users’ own time, complete program accreditation assessment and monitoring online, develop tailored action plans, and receive email reminders and prompts to complete action.

**Conclusions:**

A Web-based alcohol management approach to support sports clubs in the implementation of recommended alcohol management policies appears both feasible and acceptable. Future research should aim to determine if such intended use leads to actual use and club implementation of alcohol management policies.

## Introduction

Worldwide, approximately 3.3 million deaths and over 200 diseases and injuries are attributable to excessive alcohol consumption [1]. High levels of alcohol consumption and alcohol-related harm have been reported among players and supporters of community level, nonelite sports clubs [2,3]. For instance, in New Zealand nonelite sportspeople have reported higher levels of harmful alcohol consumption (51%) than nonsports people (31%) [4], and nonelite Gaelic football and hurling players in Ireland have reported higher levels of alcohol consumption (32%) compared to a national representative sample of men of a similar age (15%) [3]. Similarly, reported levels of alcohol consumption of nonelite football players in Australia are between 4 and 9 times the recommended level of alcohol per drinking session [2,5].

In many nations, nonelite community sports clubs provide opportunities for organized sports participation for children and adults. A number of characteristics of such community sports clubs make them an attractive setting to address risky alcohol use and alcohol-related harm among players and supporters. First, sports clubs provide access to large numbers of players, spectators, and officials [6]. For instance, in England between 2015 and 2016, 15.83 million people 16 years or over (36.1%) engaged in sport at least once a week [7] and, in Australia, between 2013 and 2014, approximately 5.2 million Australians aged 15 years and over (28%) were involved with organized sport and physical activity [8]. Second, despite community sports clubs being required to adhere to liquor licensing laws regarding responsible service of alcohol [9-11] to reduce the risk of excessive alcohol consumption and alcohol-related harm [12], such adherence is poor [13,14]. For example, a study conducted in 87 community football clubs in New South Wales (NSW), Australia, found 32% did not have all bar staff trained in the Responsible Service of Alcohol (RSA), 38% conducted high-risk drink promotions, and 35% allowed bar staff to consume alcohol while on duty [14]. Third, research has found that community sports clubs are amenable to support to improve alcohol management to reduce alcohol use and harm occurring at these venues [15,16].

Studies have demonstrated that implementing comprehensive alcohol management policies (eg, responsible service of alcohol and inhibiting alcoholic drink promotions) in licensed venues can reduce harmful alcohol use in such premises [17,18]. Similarly, evidence suggests that the implementation of comprehensive alcohol management policies by sporting clubs can reduce excessive alcohol consumption by members in these venues and their risk of alcohol-related harm [19]. For example, in a randomized controlled trial (RCT) of an alcohol management accreditation program in community football clubs in Australia, clubs received face-to-face and telephone-based support to implement a suite of policies to reduce the risk of excessive alcohol use [14]. Postintervention, a significantly greater proportion of intervention clubs (88%) implemented alcohol management policies compared to control clubs (65%), and a significantly lower proportion of club members from intervention group clubs engaged in risky alcohol consumption at the club compared to control clubs [14].

The ability to modify the policies of service delivery organizations is suggested to be enhanced by the use of a variety of evidence-based implementation change strategies such as audit and feedback, consensus processes, training and resource provision [20]. In an RCT of an alcohol management accreditation program in community football clubs in Australia, a number of such strategies were provided on a face-to-face and telephone basis to support the implementation of alcohol management policies. The delivery of implementation support in this manner can represent a logistical and resourcing challenge when a large number of sites (eg, sports clubs, hospitals, schools) are involved and when such sites are geographically dispersed or remote [21]. Computer or Web-based delivery of policy change strategies have become increasingly common and have been shown to be efficacious in improving implementation of evidence-based policies in some settings, such as primarily health care services [22]. For example, in health care settings, Web-based delivery of training, audit, and feedback strategies have been reported to be effective in modifying the provision therapeutic interventions by clinicians [23].

Web-based programs can be delivered at relatively low cost to large numbers of sports clubs and may represent a potential means of overcoming the logistical challenges of scaling up evidence-based alcohol management policies in this setting. Internet coverage is almost universal in high income countries such as Australia [24], extending into rural and remote geographic locations, enabling the provision of Web-based support to clubs located in these areas. Furthermore Web-based programs provide consistent, standardized delivery of content, can tailor content to specific needs of users, and have the functionality to incorporate evidence-based techniques to support implementation (eg, performance monitoring and feedback, action planning and goal setting, and social comparison) [25-29]. While Web-based programs have been used to support implementation or support quality improvement initiatives in other settings, such as hospitals, general health care [22,30], and schools [31-33] we are not aware of evaluations of such initiatives in the sporting club environment.

Despite the potential of Web-based programs, the benefits of such programs are often encumbered by low user engagement and uptake [30,34]. Given this risk of nonutilization, assessment of the feasibility and acceptability of Web-based technologies to end-users has been recommended before significant investments in the development of Web-based programs are initiated [35,36]. The Technology Acceptance Model (TAM) is a validated, widely used, and recommended tool to pre-assess factors associated with end-user intention to use a Web-based program (perceived ease of use and perceived usefulness) [36,37] and to assess the potential use and impact of Web-based technologies. Given the lack of empirical studies examining the use of Web-based programs by sports clubs and club administrators generally and of the potential of such programs to support club implementation of alcohol management policies specifically, a study was undertaken to assess the following:

1. Current access to and use of the Web and electronic devices by community sports club administrators
2. Administrator perceptions regarding the usefulness and ease of use of a hypothetical Web-based support program to support club implementation of alcohol management policies and their intention to use such a program
3. Factors associated with intention to use a Web-based alcohol management support program
4. Specific features of such a program that sports club administrators would find useful

## Methods

### Design and Setting

A cross-sectional survey of club administrator representatives from community football clubs was conducted in the state of NSW, Australia. Clubs were based in major city, regional, and rural communities.

### Participant Eligibility and Recruitment

#### Football Clubs

Participating clubs were community level, nonelite football clubs across the 4 major Australian football codes: Rugby League, Rugby Union, Soccer/Association football, and Australian Football League. Eligible clubs had players over the legal drinking age (18 years of age and over), were a nonelite community sports club (defined as clubs not involved with a major national or state level league or competition), had over 40 members, and held a current liquor license enabling sale of alcohol at the sports club and were currently participating in an existing alcohol management program delivered on a face-to-face basis (Good Sports) [38]. Additionally, clubs had participated in an RCT conducted between 2009 and 2012, which evaluated the effectiveness of a face-to-face alcohol management program to support clubs to implement alcohol management policies [14]. A full description of the intervention has been published [14]; in short, the intervention included hard copy resources, club committee engagement, and face-to-face monitoring and feedback for each intervention club.

#### Football Club Administrators

A senior club administrator (eg, president, vice president, or secretary) of eligible clubs was identified from club records and sent a study information letter and invited to participate in the study on behalf of the club.

### Data Collection Procedures

An expert advisory group with representation from community sports clubs (senior club administrators), health promotion practitioners, implementation and behavioral scientists, and experts in organizational change informed the development of a computer-assisted telephone interview (CATI) survey, based on previously implemented surveys in sports clubs and other community settings [14,39]. The CATI was piloted on a subsample of community football club administrator representatives to ensure survey language and length was appropriate. Surveys were conducted by trained interviewers in the Australian winter football season (June-August) of 2015. The average length of the survey was 40 minutes.

### Measures

#### Football Club and Club Administrator Characteristics

Club administrators were asked to report the club’s home ground postcode, football code (Rugby League, Rugby Union, Soccer/Association football, or Australia Football League), the number of senior (18 years of age and over) and junior (under 18 years of age) teams registered with the club, their current role within the club, the time (in years) they had been in that role, their age, and their gender.

#### Football Clubs’ Current Use of the Web and Electronic Devices

Club administrators were asked to report whether they had access to the Internet and whether they used electronic devices (eg computers, iPads/tablets, smartphones, laptops, televisions, and smartboards) to complete specific club-related tasks (at any location), including membership and player registration, game scheduling, managing club finances/book keeping, communicating with members, committee administration tasks, administration fundraising, and other events.

#### Perceived Usefulness, Ease of Use, and Intention to Use a Web-Based Alcohol Management Program

The TAM is a validated instrument for prospectively assessing end-user intention to use Web-based programs [36,37]. TAM consists of 3 primary constructs (behavioral intention, perceived ease of use, and perceived usefulness) for which a meta-analysis of 88 studies has reported high internal consistency among each construct (Cronbach alpha score of >0.8) [40]. Derived from the theory of reasoned action, TAM postulates that behavioral intention is linked to actual behavior [36,41]. A systematic review of the TAM literature supports this, with a positive correlation of association between behavioral intention and actual use of technology found for 90% of included studies [35].

A total of 11 items from TAM were adapted for relevance to the sports club setting. A hypothetical Web-based program was described to club administrators via the CATI to determine clubs’ perceived usefulness, ease of use, and intention to use the program to support club implementation of recommended alcohol-management policies. The program was described to club administrators as able to monitor their progress of alcohol policy implementation, provide tailored feedback on their level of implementation, send prompts and reminders for required tasks, and provide unrestricted access to information and resources. Similar adaptation of the TAM has been employed in other community settings [39]. Club administrators were asked to rate on a 7-point scale (1=strongly disagree, 4= neither agree nor disagree, and 7=strongly agree) the perceived usefulness of a Web-based alcohol management program (4 items), the perceived ease of use of such a program (4 items), and their intention to use such a program (3 items) (see Multimedia Appendix 1 for modified TAM questionnaire, including a description of the hypothetical Web-based program).

#### Specific Features of an Alcohol Management Web-Based Program That Sports Club Administrators Would Find Useful

A total of 10 questions were used to assess the perceived usefulness of specific Web-based program features to support club implementation of an alcohol management program. Responses were recorded on a 7-point scale (1=strongly disagree and 7=strongly agree). Assessed program features were ability to complete program requirements in users’ own time, ability to complete program accreditation assessment and monitoring online, development of tailored action plans, email reminders and prompts to complete action items, access to support tools and resources, access to training and educational videos or interactive activities, program support via email or live chat, ability to communicate with club members (email), ability to communicate with other sports clubs in the program, and program-related peer comparison.

### Statistical Analysis

Statistical analyses were conducted in SAS version 9.3 statistical software (SAS Institute Inc). Clubs were grouped by football code (Rugby League, Rugby Union, Soccer/Association football, and Australian Rules Football), and classified according to geographical location (major city or inner regional/outer regional) using the Australian Standard Geographical Classification (ASGC) based on the Accessibility/Remoteness Index of Australia score (ARIA) and size (small [≤10 teams] or large [>10 teams]). All statistical tests were 2-tailed with an alpha of 0.05.

For aim 1, simple descriptive statistics were used to describe sports club administrators’ access to and use of the Web and electronic devices to undertake club tasks. For aim 2, similar to other TAM studies [39,42], the mean score and standard deviation for each TAM construct was calculated. The internal consistency of each TAM construct was assessed using Cronbach alpha.

For aim 3, again similar to other TAM studies [39,42], the 3 TAM constructs were dichotomized into a score of 1 (strongly disagree) to 5.9 (slightly agree) or 6 (agree) to 7 (strongly agree). By choosing this cut-point, the results allow for a clinically meaningful interpretation, as the median score of the constructs were used. Additionally, this dichotomized score differentiates between those who disagree or only slightly agree to those who have full to strong agreement with the items examined within the TAM constructs. Fisher’s exact test was used to test the significance of association between club administrator intention to use the Web-based program (1.0-5.9 [low intention] vs 6.0-7.0 [high intention]), club geographic location, size, administrator age (≤50 years vs >50 years), use of electronic device for club tasks (≥1 device vs no devices), access to the Internet when undertaking club tasks (yes vs no), perceived ease of use of the Web-based support program (construct mean score dichotomized: 1.0-5.9 [low perceived ease of use] vs 6.0-7.0 [high perceived ease of use]), and perceived usefulness of the Web-based support program (construct mean score dichotomized: 1.0-5.9 [low perceived usefulness] vs 6.0-7.0 [high perceived usefulness]). For aim 4, descriptive statistics were used to summarize the features of a Web-based support program that sports clubs administrators agreed or strongly agreed that they would find useful to support implementation of recommended alcohol management policies.

### Ethics Approval

Ethics approval was obtained from The University of Newcastle, Human Research Ethics Committee (H-2008-0432).

## Results

### Football Club and Club Administrator Characteristics

A total of 73 community sporting clubs were approached to participate in the study, of which 63 consented to participate (86%). Of these 63, 17 were ineligible and 46 completed the survey (73% of eligible). Of the participating clubs, the largest proportion were Rugby League clubs (15/46, 33%) and Rugby Union clubs (14/46, 30%), the majority of clubs (38/43, 88%) were located in major cities, and just over half the sample (24/46, 53%) was classified as large clubs. Most club administrator representatives held the role of club president (15/46, 33%) or secretary (14/46, 30%) and were male (39/46, 85%), with a mean age of 48 years and a mean number of 4.3 years in that position (Table 1).

### Football Clubs’ Current Use of and Access to the Web and Electronic Devices

Most (98%) football club administrators reported current use of electronic devices for club-related tasks and all reported having access to the Web when undertaking these tasks. The proportion of clubs that reported undertaking specific tasks using electronic devices is reported in Table 2.

**Table 1:** Football club and club administrator representative characteristics (N=46).

Characteristics			Number
**Football club characteristic**			
	**Football code**		
		Australian League Football, n (%)	6 (13)
		Rugby League, n (%)	15 (33)
		Soccer/Association football, n (%)	11 (24)
		Rugby Union, n (%)	14 (30)
	**Geographical region^a^**		
		Major city, n (%)	38 (88)
		Inner/outer regional, n (%)	5 (12)
	**Club size**		
		Small (≤ 10 teams), n (%)	21 (47)
		Large (>10 teams), n (%)	24 (53)
**Club administrator characteristics**			
	**Club role**		
		President, n (%)	15 (33)
		Vice President, n (%)	4 (9)
		Secretary, n (%)	14 (30)
		Treasurer, n (%)	4 (9)
		Coach, n (%)	1 (2)
		Committee member, n (%)	2 (4)
	Time in club role, years, mean (SD)		4.3 (3.2)
	Age, years, mean (SD)		49 (9.64)
	Gender, male, n (%)		39 (85)

^a^N=43.

**Table 2:** Proportion of clubs reporting the use of the electronic devices to undertake specific club-related tasks (N=46).

Club-related task	n (%)
Membership and player registration	45 (98)
Game scheduling	38 (83)
Managing club finances/bookkeeping	44 (96)
Communicating with members	45 (98)
Committee administration tasks	45 (98)
Administration fundraising and other events	44 (96)

**Table 3:** Football clubs perceived usefulness, ease of and intention to use a Web-based program to support implementation of recommended alcohol management policies.

TAM^a^ items and constructs		Mean (SD)
**Perceived usefulness**		
	I would find Good Sports online useful in helping my club implement Good Sports policies.	6.30 (0.70)
	Using Good Sports online would improve my clubs PERFORMANCE in implementing Good Sports policies.	6.20 (0.96)
	Using Good Sports online would increase my clubs PRODUCTIVITY in implementing Good Sports policies.	6.07 (1.04)
	Using Good Sports online would help enhance the EFFECTIVENESS of my club in implementing of Good Sports policies.	6.11 (0.97)
	*Overall usefulness of Good Sports online*^b^	6.17 (0.85)
**Perceived ease of use**		
	My interaction with Good Sports online would need to be clear and understandable.	6.33 (0.97)
	Interacting with Good Sports online is not likely to require a lot of my mental effort.	5.83 (1.32)
	I would find Good Sports online easy to get it to do what I want it to do.	5.89 (1.16)
	I would find Good Sports online easy to use.	5.96 (1.09)
	*Overall perceived ease of use of Good Sports online*^b^	6.00 (0.99)
**Perceived intention of use**		
	Assuming I had access to Good Sports online, I INTEND to use it.	6.24 (0.92)
	Given that I had access to Good Sports online, I PREDICT that I would use it.	6.24 (0.90)
	If Good Sports online was currently available, I would PLAN to use it in the next 12 months.	6.28 (0.91)
	*Overall intention to use Good Sports online*^b^	6.25 (0.87)

^a^TAM: Technology Acceptance Model.

^b^Cronbach alpha score of >0.9 for each TAM construct: usefulness, perceived ease of use, and intention to use a Web-based program.

### Perceived Usefulness, Ease of Use, and Intention to Use a Web-Based Alcohol Management Implementation Program

Table 3 presents the mean score and standard deviation for each individual TAM question and overall for each of the 3 TAM constructs. Internal consistency for each construct was high, with a Cronbach alpha score of >0.9 for each of the 3 TAM constructs. For all 11 items within the TAM domains club administrators had high scores, with a mean score of 5.83 or greater (max 7) for all items. The perceived usefulness of a Web-based program to help sports clubs implement recommended alcohol management policies was high; with an overall mean construct score of 6.17 (SD 0.85). Similarly, the perceived ease of use (construct mean 6.00, SD 0.99) and intention to use such a Web-based support program were high (construct mean 6.25, SD 0.87). A total of 89% of clubs reported a high behavioral intention to use a Web-based alcohol management program.

### Club and Administrator Characteristics and Perceptions Associated With Perceived Intention to Use a Web-Based Alcohol Management Program

Table 4 presents the results of tests of association between club and administrator characteristics and perceived ease of use, perceived usefulness, and perceived intention to use a Web-based program to support implementation of alcohol management policies. The characteristics that were found to be positively associated with high intention to use a Web-based alcohol management program were perceived ease of use (*P*=.02) and perceived usefulness of the program (*P*=.03). Club size was found to be positively significantly associated with high intention to use such a program (*P*=.02).

**Table 4:** Associations between club and administrator characteristics and perceptions and intention to use a Web-based alcohol management program.

Characteristics and perceptions		Intention to use^a^		Relative risk for high intention of use RR (95% CI)	Fisher’s exact *P* value
		Score of 1.0-5.9 N=5 n (%)	Score of 6.0-7.0 N=41 n (%)		
**Geographical region^b^**					.48
	Major city	4 (11)	34 (89)	1.1 (0.8-4.4)	
	Inner/outer regional	1 (20)	4 (80)	—	
**Club size**					.02
	Small	5 (24)	16 (76)	0.8 (0.5-0.9)	
	Large	0 (0)	24 (100)	—	
**Club administrator age**					.65
	50 years or less	2 (8)	23 (92)	1.1 (0.8-1.5)	
	Over 50 years	3 (14)	18 (86)	—	
**Current use of electronic devices for club tasks**					1.00
	1 or more devices	5 (11%)	40 (99%)	0.9 (0.7-30.8)	
	No devices	0 (0%)	1 (100%)	—	
**Access to internet when undertaking club tasks**					1.00
	Yes	5 (11%)	40 (89%)	—	
	No	0 (0%)	0 (0%)	—	
**Perceived ease of use^a^**					.02
	1.0-5.9	4 (31%)	9 (69%)	—	
	6.0-7.0	1 (3%)	32 (97%)	1.4 (1.0-2.9)	
**Perceived usefulness^a^**					.03
	1.0-5.9	3 (37%)	5 (63%)	—	
	6.0-7.0	2 (5%)	36 (95%)	1.5 (1.0-6.8)	

^a^Score of 1.0-5.9 indicates response to statements of strongly disagree to slightly agree, and score of 6.0-7.0 indicates response to statements of agree and strongly agree.

^b^Clubs categorized using the Australian Standard Geographical Classification (ASGC), which classifies remoteness based on sports clubs postcodes matching the Accessibility/Remoteness Index of Australia (ARIA) score.

### Specific Features of an Alcohol Management Web-Based Program That Sports Club Administrators Reported Would Be Useful

Overall, there was a high level of agreement regarding the usefulness of each of the proposed program features. All club administrators agreed or strongly agreed that the ability to complete program requirements within their own time would be a useful feature. Greater than 90% of club administrators agreed or strongly agreed that it would be useful for the program to enable completion of program accreditation assessment and monitoring online (96%), develop tailored action plans (96%), send email reminders and prompts to complete actions (96%), provide access to support tools and resources (96%), include program support (email or live chat) (94%), and provide program-related peer comparison (91%). A high level of support was evident for access to videos or interactive activities for training and education (83%), an ability to communicate with club members by email (78%), and an ability to communicate with other sports clubs in the program (70%).

## Discussion

### Principal Findings

This is the first study to assess the feasibility and acceptability of a Web-based program to support the implementation of alcohol management policies by community sports clubs. There was universal access to the Web and use of electronic devices when undertaking club-related tasks among clubs. The vast majority (89%) of club administrators reported high behavioral intention to use a Web-based program to support their club’s implementation of recommended alcohol management policies. Furthermore, both perceived usefulness of a Web-based alcohol management program and its perceived ease of use were positively associated with intended use. The findings suggest that there is considerable potential for a Web-based program to support sports clubs in the implementation of recommended alcohol management policies and in doing so to make a contribution to reducing alcohol-related harm in this setting and the community at large.

The study findings are comparable to similar studies conducted in other settings. For example, high intention to use Web-based programs to support implementation of health-related programs have been reported in childcare services [39], health care centers [43], and other community settings [42]. Furthermore, significant associations between perceived ease of use or perceived usefulness of Web-based programs with their intended use has been consistently reported across settings [37,39,43]. To ensure a high level of perceived ease of use and usefulness, such findings underscore the importance of program features designed to address barriers to engagement (eg, assimilation and personalization reduction) [27,44] and the importance of formative research with end-users to ensure the development of useful programs that meet their needs.

In this study, larger clubs had a significantly higher level of intended program use (100%) compared to smaller clubs (76%). This may be due to greater complexity of managing alcohol use in larger clubs, requiring greater workforce, resources, and infrastructure to manage. Potentially, the use of Web-based programs to coordinate the implementation of alcohol management policies in these settings may be perceived as of greater benefit. Similarly, as in licensed venues, sports clubs experience a high number of alcohol-related incidents [45,46], Web-based support to reduce such alcohol-related harm may be more salient among administrators of larger clubs. Nonetheless, the findings suggest that Web-based support may be less effective in supporting improvements in alcohol management in smaller clubs. Future research to identify alternate and adjunctive models of support for such clubs appears warranted.

### Strengths and Limitations

The results of this study should be considered with respect to its strengths and limitations. The strengths of this study include the application of a validated tool [40] to assess intended use of a hypothetical Web-based program, strong internal consistency across TAM constructs, and complete data for all participants. On the other hand, while club administrators are those most likely to be coordinating and overseeing the introduction of a Web-based program to support the implementation of recommended alcohol management policy, it is possible that they may not be representative of all individuals involved with clubs that may at times use such a program, such as volunteers and other staff. Clubs in this study were randomized into either the intervention or control arm of a face-to-face alcohol management trial between 2009 and 2012 [14], thus some of the clubs would have received intensive intervention support throughout this time. Therefore, it should be noted that previous intervention clubs within this group may be more likely to find it feasible and acceptable to use a Web-based program to support alcohol policy implementation. In addition, findings from this study may not be able to be generalized to groups outside of the study sample, such as other sporting codes or other community organizations. Finally, although there is empirical evidence [35] to show that intended use of a program is linked to actual use, actual rates of use of a Web-based program to support the implementation of recommended alcohol management policies among participants in this setting has not been reported.

### Conclusion

Further studies are required to determine if sports clubs will actually use such a program if it existed and to show whether such a program has the intended effect of supporting clubs to implement recommended alcohol management policies.

## Appendix

Multimedia Appendix 1 Modified Technology Acceptance Model questionnaire (administered via computer-assisted telephone interview).

## Abbreviations

ARIA: Accessibility/Remoteness Index of Australia
ASGC: Australian Standard Geographical Classification
CATI: computer-assisted telephone interview
NSW: New South Wales
RCT: randomized controlled trial
RR: Relative Risk
RSA: Responsible Service of Alcohol
TAM: Technology Acceptance Model
