# Supplementary material for: The Feasibility and Acceptability of a Web-Based Alcohol Management Intervention in Community Sports Clubs: A Cross-Sectional Study
Source: JMIR Res Protoc. 2017 Jun 30;6(6):e123. doi: 10.2196/resprot.6859 (PMC5511365; doi:10.2196/resprot.6859)
Supplement: Multimedia Appendix 1 [file resprot_v6i6e123_app1.pdf]

Appendix 1: Modified TAM questionnaire (administered via computer assisted telephone interview)

Information screen for interviewer:
The next questions are about an online mode of the Good Sports program, which I will refer to as 'Good Sports online'. We are interested in your opinion about this new mode.

Good Sports online will include:
Online completion of accreditation level reviews and level 3 monitoring reports, tailored action plans, information and resources to support clubs in the Good Sports program, and email prompts and reminders.

Please remember that there are no right or wrong answers - your opinions will help to ensure that such a program would be useful, relevant and practical for use in sporting clubs.

When answering the following questions, we ask you to keep the description of the program in mind. I will now read out a list of statements about the program, and ask you to rate them on a scale of 1 - 7 Where:
7 = strongly agree,
1 = strongly disagree and
4 = neither agree nor disagree.

Perceived usefulness (this heading not included in the questionnaire)

I would find Good Sports online useful in helping my club implement Good Sports practices.
1 Strongly Disagree
2 Disagree
3 Slightly disagree
4 Neither agree nor disagree
5 Slightly agree
6 Agree
7 Strongly Agree
8 Don't know [DO NOT READ]
.R Refused [DO NOT READ]

Using Good Sports online would improve my clubs PERFORMANCE in implementing Good Sports practices.
1 Strongly Disagree
2 Disagree
3 Slightly disagree
4 Neither agree nor disagree
5 Slightly agree
6 Agree
7 Strongly Agree
8 Don't know [DO NOT READ]
.R Refused [DO NOT READ]

Using Good Sports online would increase my clubs PRODUCTIVITY in implementing Good Sports practices.
1 Strongly Disagree
2 Disagree
3 Slightly disagree
4 Neither agree nor disagree
5 Slightly agree
6 Agree
7 Strongly Agree
8 Don't know [DO NOT READ]
.R Refused [DO NOT READ]

Using Good Sports online would help enhance the EFFECTIVENESS of my club in implementing of Good Sports practices.
1 Strongly Disagree
2 Disagree
3 Slightly disagree
4 Neither agree nor disagree
5 Slightly agree
6 Agree
7 Strongly Agree
8 Don't know [DO NOT READ]
.R Refused [DO NOT READ]

Perceived ease of use (this heading not included in the questionnaire)

My interaction with Good Sports online would need to be clear and understandable.
1 Strongly Disagree
2 Disagree
3 Slightly disagree
4 Neither agree nor disagree
5 Slightly agree
6 Agree
7 Strongly Agree
8 Don't know [DO NOT READ]
.R Refused [DO NOT READ]

Interacting with Good Sports online is not likely to require a lot of my mental effort.
1 Strongly Disagree
2 Disagree
3 Slightly disagree
4 Neither agree nor disagree
5 Slightly agree
6 Agree
7 Strongly Agree
8 Don't know [DO NOT READ]
.R Refused [DO NOT READ]

I would find Good Sports online easy to get it to do what I want it to do.
1 Strongly Disagree
2 Disagree
3 Slightly disagree
4 Neither agree nor disagree
5 Slightly agree
6 Agree
7 Strongly Agree
8 Don't know [DO NOT READ]
.R Refused [DO NOT READ]

I would find Good Sports online easy to use.
1 Strongly Disagree
2 Disagree
3 Slightly disagree
4 Neither agree nor disagree
5 Slightly agree
6 Agree
7 Strongly Agree
8 Don't know [DO NOT READ]
.R Refused [DO NOT READ]

Behavioural intentions to use (this heading not included in the questionnaire)

Assuming I had access to Good Sports online, I INTEND to use it.
INTERVIEWER NOTE: intend refers to planning to use the system for a specific purpose, with an outcome in mind

1 Strongly Disagree
2 Disagree
3 Slightly disagree
4 Neither agree nor disagree
5 Slightly agree
6 Agree
7 Strongly Agree
8 Don't know [DO NOT READ]
.R Refused [DO NOT READ]

Given that I had access to Good Sports online, I PREDICT that I would use it.
INTERVIEWER NOTE: predict refers to making known in advance whether you would use the system in the future, based on the information provided

1 Strongly Disagree
2 Disagree
3 Slightly disagree
4 Neither agree nor disagree
5 Slightly agree
6 Agree
7 Strongly Agree
8 Don't know [DO NOT READ]
.R Refused [DO NOT READ]

If Good Sports online was currently available, I would PLAN to use it in the next 12 months.

1 Strongly Disagree
2 Disagree
3 Slightly disagree
4 Neither agree nor disagree
5 Slightly agree
6 Agree
7 Strongly Agree
8 Don't know [DO NOT READ]
.R Refused [DO NOT READ]
